# Restoration Temperature Control through Glass Transition Temperature Modulation of Shape Memory Polymer for Thermally Switchable Adhesive

**DOI:** 10.1002/advs.202309393

**Published:** 2024-05-05

**Authors:** Han Jun Park, Minsu Kim, Jihoon Lee, Moon Kyu Kwak

**Affiliations:** ^1^ Department of Mechanical Engineering Kyungpook National University Daegu 41566 Republic of Korea

**Keywords:** dry adhesive, glass transition temperature, pick‐and‐place system, shape memory polymer, switchable adhesive

## Abstract

Shape memory polymers (SMPs) undergo changes between arbitrary shapes and programmed shapes upon exposure to specific stimulus, allowing them to restore their original shape. All kinds of external stimuli have a threshold to change the shape of the SMP. Especially, for the thermal type SMP, the critical temperature for shape restoration is typically near the glass transition temperature (*T_g_*). In this study, the controllability of the restoration temperature is analyzed by adjusting the *T_g_* of the polymer using Norland Optical Adhesive 63, which can be cured with UV irradiation. By varying the ambient temperature from 20 to 120 °C during UV exposure, *T_g_* changes ranging from 35.84 to 50.50 °C are obtained, with corresponding changes in restoration temperature. As a practical application, a thermal‐activated SMP dry adhesive is developed with programmable *T_g_* and switchable adhesion. The fabricated SMP dry adhesive exhibited strong adhesion to substrates with various surface roughness. Additionally, the shape memory effect allowed for easy detachment through shape recovery, and different adhesive performances at different temperatures are achieved by programming various *T_g_* values. Moreover, the simple manufacturing process of the SMP dry adhesive is confirmed to be suitable for continuous fabrication processes based on roll‐to‐roll methods.

## Graphical abstract

A shape memory polymer dry adhesive with a micro‐lens pattern demonstrates adjustable adhesion through the shape memory effect. It adapts to various manufacturing conditions, altering its glass temperature, and adheres effectively to rough surfaces. Continuous fabrication akin to roll‐to‐roll processing is suggested.

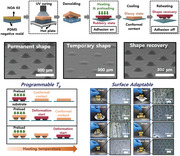

## 1 Introduction

Shape memory polymer (SMP) is a unique class of smart materials that can memorize a “permanent shape” through the shape memory effect and be fixed into a “temporary shape” by exposure to a specific stimulus such as heat, electricity, light, or a magnetic field.^[^ ^1^, ^2^, ^3^, ^4^, ^5^ ^]^ Specifically, thermally triggered SMPs, which respond to temperature changes, typically exist in a rigid state with a permanent shape at first. When heated above the glass transition temperature (*T_g_*), the elastic modulus of the SMP decreases, leading to changes induced by the movement of amorphous soft segments or crystalline hard segments in response to applied external forces.^[^ ^6^, ^7^ ^]^ Upon cooling back below the *T_g_*, the elastic modulus returns to its initial value, and even upon removal of the external force, the shape remains fixed in a deformed state (temporary shape). During this process, the cross‐linked polymer network of the SMP undergoes deformation, experiencing a glass‐rubber transition. Therefore, the *T_g_* is generally considered as the shape transition temperature of SMPs. An essential characteristic of SMPs is their ability to recover their original shape and mechanical properties upon reheating temporary shapes above the *T_g_* through a recovery effect in response to thermal stimuli. (Figure S1, Supporting Information).^[^ ^1^, ^2^, ^3^, ^4^, ^5^, ^6^, ^7^, ^8^, ^9^, ^10^, ^11^, ^12^ ^]^ SMP has shown immense potential in various applications such as biomedical devices, aerospace, and robotics due to its unique ability to switch between different shapes on‐demand.^[^ ^5^, ^13^ ^]^ One of the most promising applications of SMP is the development of transfer systems using its shape memory effect to achieve high precision pick‐up and release of fragile substrates.^[^ ^3^, ^5^, ^6^, ^11^, ^14^, ^15^ ^]^

Transfer system is a key process in the manufacturing of displays and semiconductors, which requires the ability to transfer objects with various surface profiles.^[^ ^16^, ^17^, ^18^, ^19^, ^20^, ^21^, ^22^, ^23^ ^]^ With the increasing demand for substrates that are both thinner and larger, the need for efficient transfer systems is more crucial than ever. To achieve high efficiency in the pick‐and‐place system, a strong adhesion force is required for picking‐up, as well as a weak adhesion force for placing.^[^ ^18^, ^19^, ^20^, ^21^, ^22^, ^23^, ^24^, ^25^, ^26^ ^]^ This necessitates an adhesion switching mechanism that can easily switch between these two adhesion forces. Traditionally, this has been accomplished through vacuum chuck systems, friction or interlocking systems, or chemical‐based wet adhesive systems.^[^ ^18^, ^20^, ^21^, ^23^, ^24^, ^25^, ^26^ ^]^ However, these methods are limited by their complexity and equipment requirements, and the adhesion force may not be weak enough for efficient transfer.^[^ ^18^, ^20^, ^23^ ^]^ Recently, studies have focused on dry adhesives, which utilize van der Waals interactions to achieve the required adhesion switching.^[^ ^24^, ^25^, ^26^ ^]^ These adhesives offer significant advantages during the picking‐up process, leveraging their low cost, chemical resistance, and mechanical durability, and are rapidly being commercialized in the pick‐and‐place system industry.^[^ ^27^, ^28^, ^29^ ^]^ Inspired by the microstructures of gecko lizards or octopuses, these adhesives mimic their surface properties, resulting in adhesion forces of over 20 N cm^−2^.^[^ ^28^, ^29^, ^30^, ^31^ ^]^ However, during the placing process, the high pull‐off strength may require additional effort to detach thin or fragile substrates, presenting a challenge in achieving stable detachment during the adhesion switching process.^[^ ^24^, ^25^, ^26^, ^27^, ^28^ ^]^

Another alternative strategy proposed for achieving strong adhesion and stable detachment is the utilization of SMP. Several studies have suggested various strategies using SMP to design pick‐and‐place systems. For instance, a double‐layer structure composed of an SMP layer, and a thin adhesive layer has been proposed to effectively induce switching by self‐peeling.^[^ ^32^ ^]^ Furthermore, surface microstructures, such as micro‐tips, micro‐wedge arrays, and gecko‐mimicking systems, have been introduced to enhance the transfer performance of SMP‐based dry adhesives efficiently.^[^ ^6^, ^14^, ^15^, ^33^, ^34^, ^35^, ^36^, ^37^, ^38^, ^39^, ^40^ ^]^ The Systems introduced in previous studies have implemented thermal deformation effects to achieve high adhesion forces during picking‐up and safely place substrates with low adhesion forces due to the recovery effect of microstructures. Despite their potential advantages, the current manufacturing process of SMP‐based adhesives remains time‐consuming. This is primarily attributed to the necessity for multiple material combinations to construct the double‐layer structure and the prolonged thermosetting time required during fabrication. Additionally, previous studies provided valuable insights into transfer systems using SMP but lack detailed descriptions, particularly regarding the comparison of adhesive performance according to the *T_g_*. To explore the practical application of SMP‐based transfer systems in the industry, it is essential to demonstrate their suitability for use across various temperature ranges and confirm the continuous fabrication capability of adhesive pads.

In this study, we report the design and development of a novel SMP dry adhesive for highly reversible heat‐actuated adhesion switch systems using Norland Optical Adhesive 63 (NOA 63). This material is a commercially available resin, which is a mercaptoester‐type UV‐curable oligomer.^[^ ^41^, ^42^, ^43^, ^44^, ^45^ ^]^ Originally developed as an optical adhesive, NOA 63 possesses excellent optical and strong mechanical properties.^[^ ^46^, ^47^ ^]^ NOA 63 as SMP contains acrylate monomer units with a mercapto‐ester groups in the polymer backbone, making it rubbery enough to conformally contact the substrate when the temperature of the urethane‐related polymer reaches *T_g_*.^[^ ^41^ ^]^ In particular, NOA 63 offers the ability to program the *T_g_* based on curing conditions, enabling rapid and straightforward manufacturing of SMP‐based adhesives via UV irradiation. Upon UV curing, NOA 63 undergoes a transition from a rubbery to a glassy state, resulting in reduced polymer chain mobility and diffusion. However, when the ambient temperature during UV curing rises, the polymer chains return to the rubbery state and residual reactions resume, increasing the *T_g_* until the final *T_g_* of the material is reached.^[^ ^48^, ^49^, ^50^ ^]^

The fabricated SMP dry adhesive demonstrated excellent adhesion performance to substrates with various surface topologies due to its ability to adapt to the rubbery state at temperatures above the *T_g_*. Moreover, by forming a micro‐lens pattern on the SMP dry adhesive's surface, it had advantages not only in adhesion but also in detachment. Due to the shape memory effect of the SMP dry adhesive allowed the micro‐lens pattern's shape recovery to detach the substrate easily. By altering the curing condition of the NOA 63, different *T_g_* values could be programmed, thus showing different adhesive performances at different temperature. Additionally, the SMP dry adhesive demonstrated excellent mechanical durability even after repeated adhesion cycle tests. For effective application, a pick‐and‐place experiment using a Peltier element was conducted, and furthermore, it was confirmed that continuous fabrication was possible by applying the easy UV curing process of NOA 63 to a roll‐to‐roll system.

## 2 Results and Discussion

### 2.1 Tunable Glass Transition Temperature

**Figure 1:**
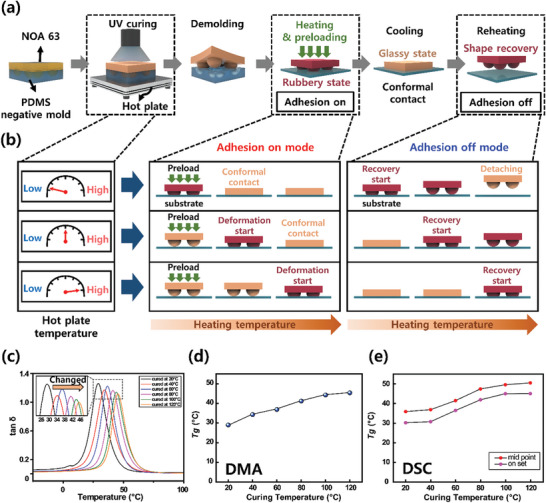
Tunable glass transition temperature of SMP dry adhesive. a) Illustration showing the process from manufacturing SMP dry adhesive to adhesion and detachment. b) Illustration of the glass transition temperature change and the resulting adhesion trend at different hot plate temperature (ambient temperature) settings during UV curing. c) Graph of tan δ from DMA measurement results. d) Graph of glass transition temperature change according to ambient temperature during UV irradiation, derived through DMA measurement and e) DSC measurement. (The ambient temperature during UV irradiation is 20, 40, 60, 80, 100, and 120 °C).

In SMP, the *T_g_* is the reference point for thermal deformation and recovery. Therefore, by using NOA 63 as a shape memory polymer and controlling the ambient temperature during the curing process, *T_g_* can be appropriately adjusted to change the temperature at which it can be transformed or recovered to the desired shape. The entire process from fabrication to use of SMP dry adhesive, including the *T_g_* modification method using NOA 63 as SMP, is shown in **Figure** **1**a. NOA 63 was poured onto the negative polydimethylsiloxane (PDMS) mold with a micro‐lens pattern and placed on a hotplate for 10 min while being cured at a UV wavelength of 365 nm. Finally, the cured SMP dry adhesive was carefully demolded from the PDMS negative mold (Figure S2a, Supporting Information). The photograph of the fabricated sample is shown in Figure S2b (Supporting Information).

The reason for heating the ambient temperature while exposing it to UV is to program the glass transition temperature of NOA 63. The principle behind modulating the *T_g_* of NOA 63 is generally determined by the chemical and physical properties of the polymer, which can be interpreted based on reaction kinetics. *T_g_* is influenced by changes in the polymer's molecular structure. As mentioned, NOA 63 contains acrylate monomers, some of which are reported to include mercapto‐ester groups that participate in the photopolymerization reaction, forming polymer chains and creating cross‐links.^[^ ^41^, ^48^, ^49^, ^50^ ^]^ The density and network strength of the resulting polymer structure play a crucial role in determining the *T_g_* of the cured material. By adjusting the ambient temperature during UV irradiation, the speed of the photopolymerization reaction and the formation of the cured polymer structure can be altered. Photopolymerization leads to the transition from a rubbery to a glassy state, reducing the mobility of polymer chains and significantly limiting reaction rates. Setting a higher ambient temperature allows some polymer chains to revert to the rubbery state, resuming residual reactions. Consequently, at higher temperatures, photopolymerization proceeds rapidly, enabling the formation of higher molecular weight and cross‐linked networks, thereby increasing the *T_g_* of the cured material. Conversely, at lower temperatures, photopolymerization slows down, resulting in the formation of cured materials with lower molecular weight and cross‐link levels, thus reducing the *T_g_*. Therefore, the *T_g_* of NOA 63 can be modulated by adjusting the ambient temperature during UV curing. In this research, the ambient temperature was set to 20, 40, 60, 80, 100, and 120 °C when curing NOA 63. DSC and DMA measurements were performed to observe the change in *T_g_* based on ambient temperature during UV curing. The DSC results for UV‐cured NOA63 at ambient temperatures of 20–120 °C (at 20 °C intervals) are shown in Figure S4 (Supporting Information) and Figure 1e. The definition of *T_g_* in the DSC results can be based on either the onset temperature where the heat flow starts to change or the temperature at the mid‐point of the curve's gradual change (mid‐point method).^[^ ^51^ ^]^ According to the DSC measurement results, *T_g_* is measured as 30.18, 30.74, 36.45, 41.89, 44.99, and 45.02 °C based on the onset temperature. Similarly, the *T_g_* derived through the mid‐point method is measured as 35.84, 36.83, 41.48, 47.39, 49.56, and 50.50 °C. The storage modulus and loss modulus can be obtained through DMA, and the behavior of the material in different states can be understood by the ratio of these two factors (tan δ). Therefore, the temperature at which the value of tan δ is at its maximum can be interpreted as the temperature at which the substance undergoes a change in state, and this temperature corresponds to the peak value of tan δ, which is defined as *T_g_*.^[^ ^51^ ^]^ The tan δ induced by DMA is shown in Figure 1c, and according to the results, *T_g_* was measured as 29.00, 34.30, 36.90, 41.18, 44.33, and 45.38 °C as the ambient temperature during curing increased (Figure 1d). This means that the glass transition temperature clearly increases as the ambient temperature increases during UV curing. In summary, through DSC and DMA measurements, it was confirmed that the change in glass transition temperature increases as the ambient temperature during UV curing increases.

Because of this feature of being able to control the *T_g_* of NOA 63, the SMP dry adhesive can be fabricated with different *T_g_* values by setting the ambient temperature differently during curing. In addition, utilizing NOA 63 as an SMP with shape deformation and recovery properties, it can be applied to microstructure surfaces to obtain a dry adhesive with reversible and switchable adhesion. By modifying the micro‐lens pattern, the adhesion can be switched between adhesion on mode and adhesion off mode. As a result, the temperature at which the micro‐lens pattern begins to deform varies for each *T_g_* value in the adhesion on mode. Adjusting the size and density of the micro‐lens pattern can contribute to the efficiency of the system and enable optimization. However, for the purpose of comparing adhesion performance through glass transition temperature modulation in this study, we standardized the pattern to a diameter of 200 µm and a height of 90 µm. As shown in Figures 1b and 3d, when the ambient temperature is set low while UV is irradiated, the *T_g_* is programmed to be lower than when set at a high temperature, causing the SMP dry adhesive to change to a rubbery state at a lower temperature range and thus transform into a temporary shape earlier. In other words, it can conformally contact with the substrate at a lower temperature range than when *T_g_* is programmed high. Similarly, in the adhesion off mode, the temperature at which the shape begins to be restored varies. The SMP dry adhesive samples produced with a high *T_g_* (when the hotplate temperature was set high) begin to recover their shape at a higher temperature range than those produced with a low *T_g_*. When gradually raising the temperature to detach the adhered substrate, the sample with a lower *T_g_* will detach first, and the sample with a higher *T_g_* will detach as the temperature rises to a higher range.

### 2.2 Concept of SMP Dry Adhesive

**Figure 2:**
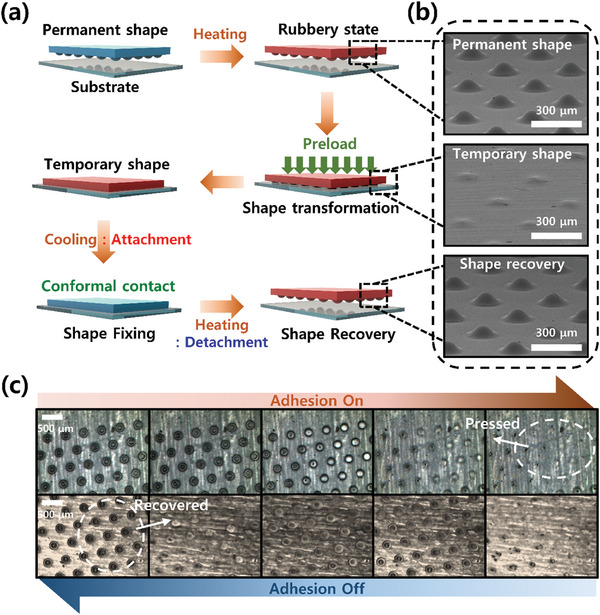
Overall concept of shape memory polymer dry adhesive. a) Mechanism of adhesion control. b) SEM images of SMP dry adhesive. Scale bar is 300 µm. c) Optical microscopic images of adhesion on mode and off mode. In the adhesion on mode, the contact area increases as the micro‐lens pattern is pressed and adheres to the substrate. In the adhesion off mode, the pressed micro‐lens pattern recovers and are separated from the substrate by minimizing the contact area.

When SMP dry adhesive is heated above its glass transition temperature, the micro‐lens pattern becomes rubbery and can be easily modified when pressure is applied. At this time, the contact area between SMP dry adhesive and the substrate increases. When the adhesive cools and returns to its glassy state, it exhibits strong bonding in the adhesion on mode. When reheated to a temperature higher than *T_g_*, the deformed micro‐lens pattern is recovered and the contact area with the attached substrate is reduced to be detached (adhesion off mode). As shown in **Figure** **2**a, the initial permanent shape of the dry adhesive pad minimizes the contact area with the substrate, making it impossible for the adhesive to attach. However, when pressure is applied while heating above the glass transition temperature, the surface of the adhesive pad is deformed to adapt to the substrate's surface roughness, maximizing the contact area for adhesion. When cooled below *T_g_* in this state, the SMP dry adhesive deformed along the substrate surface roughness and strongly bonded to the substrate. The process was reversible, with the SMP dry adhesive pad recovering its original shape by reheating and subsequently peeling off the substrate.

The reversible cycle characteristic of NOA 63 has also been confirmed in previous research.^[^ ^50^ ^]^ In previous research, these cycles involve repeatedly deforming into a temporary shape and recovering into a permanent shape, with three shape memory cycles measured. The results suggest that NOA 63 exhibits one‐way shape memory characteristics around its *T_g_*. This supports the reversible adhesion capability of the SMP dry adhesive presented in our study, allowing for reversible adhesion between the adhesive on mode and off mode.

This was also confirmed through optical microscopic images presented in Figure 2c, where the micro‐lens pattern is pressed along the surface of the attachment target during adhesion and recovers its initial lens shape when detached. SEM images of the SMP dry adhesive with a micro‐lens pattern of 200 µm in diameter and 90 µm in height are shown in Figure 2b. It was observed that the permanent shape dry adhesive pad was modified along the substrate surface by heat and pressure and was recovered to its original shape by reheating.

One of the notable advantages of the SMP dry adhesive is its remarkable adaptability to surfaces with a wide range of roughness, including glass, metal, prisms, paper, wood, and plastic. This adaptability allows the adhesive to generate high adhesion by conforming to various surface profiles in the adhesion on mode. Additionally, through the recovery process, the contact area can be minimized, enabling effective detachment in the adhesion off mode.

From the perspective of contact mechanics, the analysis shows that the adhesion force of the SMP dry adhesive varies with the contact area. If the material properties, size, height, and density of microstructures are assumed to be the same, the equation for the adhesion force *F* can be expressed as

(1) $$F\;=\;-2\pi R\frac{A}{12\rho h^{2}}$$

where *A* is the contact area, *h* is distance from the surface of a semi‐infinite body, ρ is molecule number density and *R* is effective particle radius.^[^ ^52^ ^]^ Hence, to enhance the adhesion force of the SMP dry adhesive, increasing the contact area is a crucial parameter. In summary, the contact area is an important parameter that determines the adhesion force, and the equation provides a quantifiable relationship between the two. These findings suggest that controlling the contact area could lead to further enhancements in the adhesion force and expand the potential applications of SMP dry adhesive.

### 2.3 Adhesion Performance

**Figure 3:**
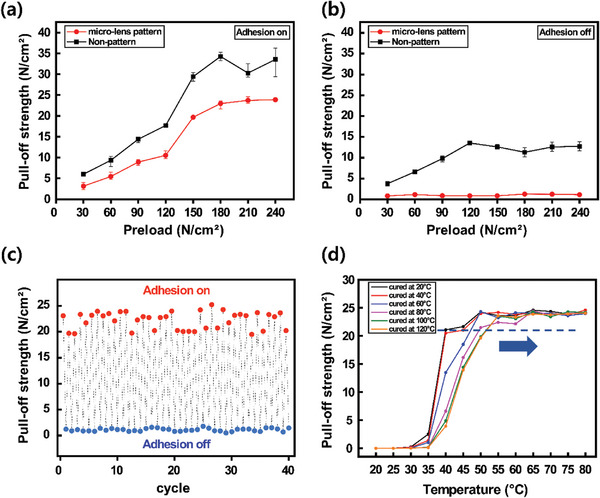
Adhesion performance of SMP dry adhesive. a) Pull‐off strength in adhesion on mode of non‐pattern pad and micro‐lens SMP dry adhesive. b) Pull‐off strength in adhesion off mode of non‐pattern pad and micro‐lens SMP dry adhesive. c) Durability of SMP dry adhesive. Pull‐off strength measured after 40 adhesion on‐off. d) Pull‐off strength according to the temperature of SMP dry adhesives by ambient temperature during UV irradiation. The pull‐off strength was measured with a preload of 240 N cm^−2^.

The micro‐lens pattern of SMP dry adhesive plays a crucial role in controlling adhesion. To investigate the effect of micro‐lens pattern on adhesion control, the pull‐off strength by preload was measured for both non‐pattern NOA 63 pads and micro‐lens pattern SMP dry adhesive. As shown in **Figure** **3**a, the pull‐off strength increases with increasing preload in adhesion on mode. Although the non‐pattern pad has a higher pull‐off strength of 34 N cm^−2^ or more, the micro‐lens pattern SMP dry adhesive also shows a sufficiently strong adhesive force of 23 N cm^−2^ or more. Since the non‐pattern pad is advantageous in surface deformation, it has a larger contact area, and thus have high pull‐off strength. Nevertheless, the advantage of micro‐lens pattern is revealed in the adhesion off mode. Figure 3b shows that the pull‐off strength of the micro‐lens pattern SMP dry adhesive is almost removed, while the non‐pattern pad has a pull‐off strength of up to 13 N cm^−2^. This suggests that detaching the substrate from the non‐pattern pad may cause damage due to the residual adhesion.

Additionally, the durability of SMP dry adhesive with micro‐lens pattern was examined. The pull‐off strength was measured by repeating adhesion on mode and adhesion off mode 40 times. As a result, it was found that the pull‐off strength of 19–24 N cm^−2^ was consistently maintained in adhesion on mode, and almost all adhesion was removed in adhesion off mode (Figure 3c).

Based on the tunable *T_g_* characteristics of NOA 63, the temperature at which deformation starts can be modified to achieve a broad range of adhesive performance. Figure 3d demonstrates the results of pull‐off strength measurements for samples produced by curing SMP dry adhesive at temperatures ranging from 20 to 120 in 20 °C increments during UV curing. At a preload of 240 N cm^−2^, which is sufficient to press the micro‐lens pattern along the substrate, the adhesive was attached at temperatures ranging from 20 to 80 in 5 °C increments before being cooled to 20 °C. Each sample is in conformal contact when cooled after preloading at a temperature significantly above the *T_g_* (over 55 °C), resulting in a pull‐off strength of over 20–25 N cm^−2^. It should be noted that the *T_g_* of SMP dry adhesive also increases as the ambient temperature rises during UV irradiation. As a result, it is confirmed that the pull‐off strength stabilizes at a higher temperature of the SMP dry adhesive UV‐cured at 120 °C than the one cured at 20 °C. This is because, as the *T_g_* increases, the SMP dry adhesive transforms into a rubbery state at a higher temperature, allowing for conformal contact to occur. Furthermore, the variation in tensile storage modulus is measured through DMA results by setting different ambient temperatures during the curing of NOA 63, to provide further support for this observation (Figure S3, Supporting Information). According to the results, samples with higher ambient temperatures exhibited a more significant decrease in tensile storage modulus at temperatures above *T_g_* compared to samples with lower ambient temperatures, and the tensile storage modulus values converged at higher values for all samples. This clearly indicates that as *T_g_* increases, the temperature at which the SMP dry adhesive becomes activated also increases.

### 2.4 Adaptability of SMP Dry Adhesive

**Figure 4:**
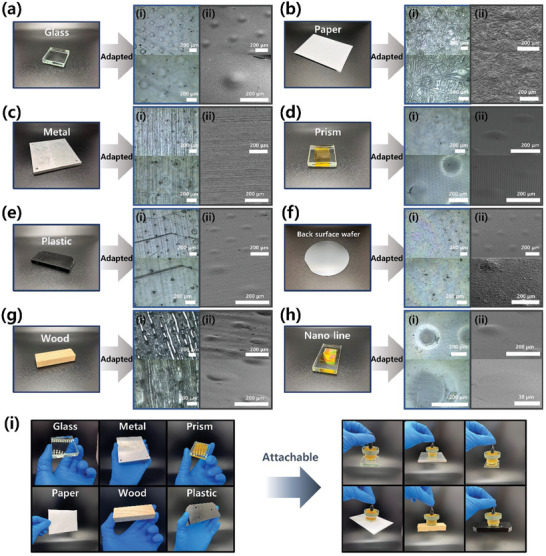
Microscopic and SEM images of the micro‐lens pattern deformed along the substrate surface of various roughness; a) Glass surface, b) Paper surface, c) Metal surface, d) Prism surface, e) Plastic surface, f) Back surface of si‐wafer, g) Wood surface h) 700 nm line surface. i) SMP dry adhesive's remarkable adaptability and lifting capability against various roughness of substrates. (i:microscopic images, ii:SEM images).

SMP dry adhesive exhibits adaptability to surfaces with varying roughness, making it a versatile adhesive for a wide range of applications. In the adhesion on mode, the SMP dry adhesive can conform not only to smooth surfaces like glass but also to rough surfaces such as wood or paper. Microscope and SEM images in **Figure** **4**a–h show the shape of the SMP dry adhesive modified along the surface of substrates with varying roughness. Heating the SMP dry adhesive on glass (RMS: 0.090 µm), metal (RMS: 0.645 µm), plastic (RMS: 0.347 µm), wood (RMS: 0.752 µm), paper (RMS: 3.012 µm), prism (RMS: 1.355 µm), back surface of wafer (RMS: 0.251 µm), and nano‐line substrate (RMS: 0.437 µm) gradually deforms the micro‐lens pattern of SMP dry adhesive along the substrate's surface, leading to increased contact area and conformal contact. The surface roughness of the substrates is shown in Figure S5 (Supporting Information), and it was confirmed that the SMP dry adhesive was properly adapted to the substrates. In the adhesion off mode, SMP dry adhesive's micro‐lens pattern is recovered and detached from the substrates, making it reusable. Figure S6 (Supporting Information) shows the recovery process of the micro‐lens pattern through the shape memory effect when SMP dry adhesive is reheated while attached to each substrate. The micro‐lens pattern gradually recovers their original shape, releasing the stored energy from deformation, and safely detaching from the substrate. This successful shape recovery process demonstrates SMP dry adhesive's potential for reusability and eco‐friendliness in adhesive applications.

### 2.5 Applications

**Figure 5:**
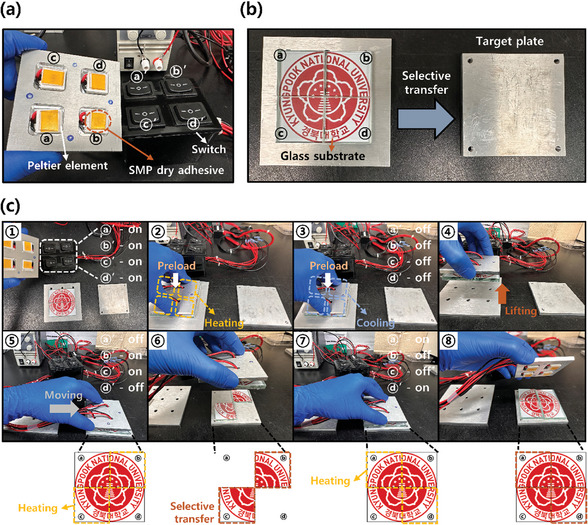
Applicability of SMP dry adhesive to pick‐and‐place system. a) Thermoelectric elements (Peltier elements) with SMP dry adhesives fixed, and a switch that can control the direction of each current. b) Glass substrate engraved with KNU mark and target plate to be transferred through selective transfer. c) Selective transfer process of SMP dry adhesive by temperature control. 2D schematic showing the sequence of separation of the glass substrates at the bottom.

To explore the practical application of SMP dry adhesive in the industry, several experiments were conducted. It was shown that the adhesive has strong adhesion to the substrate by deforming its surface and can be detached carefully through shape recovery. **Figure** **5** demonstrates the detachment of four glasses engraved with the “KNU mark” sequentially using a thermoelectric device (Peltier element). By passing an electric current (switch on) through the Peltier element, one side is heated while the other side is cooled. If the direction of the current is reversed (switch off), the heated side is cooled and the opposite side is heated. Figure 5a shows a picture of the SMP dry adhesive fixed to the four Peltier elements and each switch connected to change the current direction. Since the heating temperature is higher than the *T_g_* of the SMP dry adhesive, the adhesion can be turned on or off as desired. This experiment showed that SMP dry adhesive can be applied to the pick‐and‐place process. As shown in the Figure 5b,c, after attaching the glass substrates in the adhesion on mode, the substrate can be moved to a target plate and then detached by switching to the adhesion off mode. When the switch is turned on, the SMP dry adhesive becomes rubbery state. In this state, when the SMP dry adhesive is placed on the glass substrate with the “KNU mark” and preload is applied, the microstructure of the SMP dry adhesive surface is deformed along the substrate, and it is in adhesion on mode by turning off the switch. The lifted substrates are then transferred to the target plate and heated by operating the switches “b” and “c”, the micro‐lens patterns of b and c are recovered. Thus, the glass substrate is separated. In this way, the switch of the desired substrate can be operated and placed on the target substrate. The experimental results confirm various abilities such as adhesion switching ability, adaptability, and reversibility. Furthermore, a potential application with promising feasibility for advanced pick‐and‐place systems is proposed.

### 2.6 Possibility of Continuous Fabrication of SMP Dry Adhesive (via R2R System)

**Figure 6:**
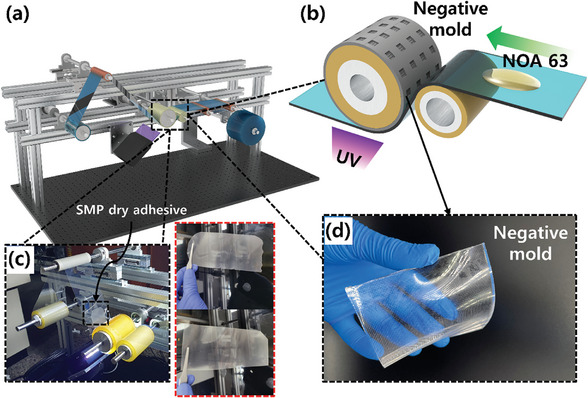
Illustration image of possibility of continuous fabrication of SMP dry adhesive through roll‐to‐roll system. a) Schematic image of the overall roll‐to‐roll equipment. b) Schematic image of the part where poured NOA 63 is UV cured in a negative mold fixed to a roll. c) Photo image of actual SMP dry adhesive fabricated by roll‐to‐roll process. d) Image of negative PDMS mold with micro‐lens pattern.

To practically apply SMP dry adhesive in industry, it is crucial to continuously fabricate the SMP dry adhesive samples. As the NOA 63 used in this research is UV‐curable material and the manufacturing process is straightforward, it is feasible to achieve continuous fabrication of the SMP dry adhesive using a roll‐to‐roll system, as demonstrated in **Figures** **6**a and S7 (Supporting Information). Previous studies have reported on the development of continuous fabrication systems utilizing NOA materials and nanoscale molds.^[^ ^53^ ^]^ In addition, it has been suggested in literature that the *T_g_* of NOA 63 can be programmed by pre‐curing at room temperature and post‐curing with UV after changing the ambient temperature.^[^ ^48^ ^]^ Therefore, the SMP dry adhesive developed in this study is a viable option for continuous fabrication in a roll‐to‐roll process, as the *T_g_* can be programmed through post‐curing. The fabrication process of SMP dry adhesive through a roll‐to‐roll system using NOA 63 is demonstrated in Figure 6b. A roll of film poured with NOA 63 passes through a negative mold where UV curing takes place (Figure 6d). Once cured, the NOA 63 is separated from the film to produce SMP dry adhesive. As seen in Figure 6c, the cured NOA 63 is rolled up on the rewinding roll, resulting in the continuous fabrication of SMP dry adhesive.

## 3 Conclusion

In this study, we successfully programmed the *T_g_* of the polymer using NOA 63, a thermal type SMP curable by UV light, and confirmed the critical temperature controllability. It was verified that the *T_g_* can be programmed to various values from ≈30 to 50 °C while changing the ambient temperature when NOA 63 is cured. Also, we developed the SMP dry adhesive that can exhibit different adhesive performance at various temperatures based on the *T_g_* setting of NOA 63. It had a remarkable pull‐off strength of ≈24 N cm^−2^ in the adhesion on mode and was adaptable to various surface topologies. Moreover, through the integration of a micro‐lens pattern on the surface, we were able to effectively reduce the pull‐off strength to 1.5 N cm^−2^ or less in the adhesion off mode, ensuring safe detachment of the substrate. The SMP dry adhesive exhibited excellent mechanical durability even after more than 40 cycle tests. Furthermore, we conducted a pick‐and‐place experiment using a Peltier element and confirmed that continuous processing was possible by applying the easy UV curing process of NOA 63 to a roll‐to‐roll system. The exceptional adhesive switching ability, durability, adaptability, and straightforward manufacturing process of the SMP dry adhesive make it a promising candidate for cutting‐edge technologies in fields that require adhesive switching, such as semiconductors or micro‐LEDs. The findings of this study indicate that the proposed SMP dry adhesive possesses strong potential for pick‐and‐place applications in a broad range of industry fields.

## 4 Experimental Section

### Design of Master Mold

The master mold with the micro‐lens pattern (diameter: 200, height: 90, spacing: 400 µm) was 3D printed using IP‐S photoresist with 1 µm resolution and the Photonic Professional GT equipment from Nanoscribe. Following the fabrication process, the master mold was subjected to surface passivation to facilitate demolding in the subsequent soft‐lithography process.

### Preparation of PDMS Negative Mold

To create the negative soft mold required for producing the SMP dry adhesive samples, the traditional soft‐lithography method was employed.^[^ ^54^, ^55^ ^]^ For this study, PDMS (Sylgard 184 kit, Dow Corning Corp., USA) was used, a silicone‐based elastomer, which was a popular choice for making molds in soft‐lithography due to its low surface energy and high durability.^[^ ^56^, ^57^ ^]^ The PDMS mixture was created by mixing the elastomer base and the curing agent in a weight ratio of 10:1, and then the mixture was degassed in a vacuum chamber. The PDMS mixture was then poured onto a 3D printed master mold and cured at 80 °C for 3 h. After the curing process, the PDMS negative mold was carefully removed from the master mold (Figure S2a, Supporting Information).

### Fabrication of SMP Dry Adhesive

To create the SMP dry adhesive, the PDMS negative mold previously prepared was used as a mold. The UV‐curable SMP NOA 63 resin (Norland Products Inc., USA) was poured onto the PDMS negative mold. The mold was then placed on a hot plate and heated while irradiating with UV at a wavelength of 365 nm for 10 min. The curing process was carried out at different temperatures, including 20, 40, 60, 80, 100, and 120 °C. Once the SMP dry adhesive was cured, it was gently removed from the PDMS negative mold (Figure 2a). The final SMP dry adhesive samples are presented in Figure S2b (Supporting Information).

### Measurement of Glass Transition Temperature

To determine the thermal and mechanical properties of NOA 63, differential scanning calorimetry (DSC) and dynamic mechanical analysis (DMA) were conducted. For DSC, a 6–7 mg sample was encapsulated in a *T_zero_* aluminum pan and heated from –50 to 200 °C at a heating rate of 10 °C min^−1^. It was then cooled back down to –50 °C and heated once again to 200 °C. DMA was conducted using a rectangular film measuring 20 mm  ×  10 mm  ×  0.65 mm^3^, loaded in force tension mode. The sample was then subjected to a temperature ramp from –50 to 150 °C at a heating rate of 3 °C min^−1^ while applying a frequency of 1 Hz. Both DSC and DMA were performed using Q2000 and Discovery DMA 850 instruments from TA Instruments, America, respectively.

### Evaluation of Adhesion Performance

The adhesion performance of SMP dry adhesive was measured using a Universal Testing Machine (UTM) (Oriental Testing M/C). The UTM was equipped with a load cell capable of measuring weight and could control movement speed and displacement through the PC. The UTM machine used in this study is shown in Figure S8 (Supporting Information). A hot plate was fixed on the bottom jig to heat the SMP dry adhesive sample. In addition, the SMP dry adhesive was fixed to a size of 1  ×  1 cm^2^ on a hot plate and a precise preload was applied. The pull‐off strength when the sample was detached from the substrate was quantified by the load cell and averaged calculated after measuring five times.

### Analysis of Surface Structure

The surface structure of the SMP dry adhesive samples was analyzed using optical microscopy (LV150L, Nikon, Japan) and scanning electron microscopy (SEM) (S‐4800, Hitachi, Japan). Prior to SEM observation, the samples were coated with a thin Pt layer of 5 nm or less using a sputtering process. The surface roughness of the substrate was measured using atomic force microscopy (AFM) (NX20, Park Systems) and confocal laser scanning microscopy (LSM700, Carl Zeiss, Germany).

## Conflict of Interest

The authors declare no conflict of interest.

## Supporting information

Supporting Information

## Data Availability

The data that support the findings of this study are available from the corresponding author upon reasonable request.
