# Supplementary material for: Restoration Temperature Control through Glass Transition Temperature Modulation of Shape Memory Polymer for Thermally Switchable Adhesive
Source: Adv Sci (Weinh). 2024 May 5;11(26):2309393. doi: 10.1002/advs.202309393 (PMC11234400; doi:10.1002/advs.202309393)
Supplement: Supplementary file 1 — Supporting Information [file ADVS-11-2309393-s001.pdf]

ADVANCED SCIENCE
Open Access

# Supporting Information

for *Adv. Sci.*, DOI 10.1002/advs.202309393

Restoration Temperature Control through Glass Transition Temperature Modulation of Shape Memory Polymer for Thermally Switchable Adhesive

*Han Jun Park, Minsu Kim, Jihoon Lee and Moon Kyu Kwak**

# Supporting Information

**Restoration Temperature Control through Glass Transition Temperature Modulation of Shape Memory Polymer for Thermally Switchable Adhesive**

*Han Jun Park, Minsu Kim, Jihoon Lee, Moon Kyu Kwak**

**Contents**

**Figure S1.** overall shape modification and recovery mechanism of shape momory polymer.

**Figure S2.** (a) Schematic illustration of the overall fabrication process of SMP dry adhesive. (b) Photo images of fabricated SMP dry adhesive. Each sample was cured at the following temperatures; (i) 20 ℃, (ii) 40 ℃, (iii) 60 ℃, (iv) 80 ℃, (v) 100 ℃, (vi) 120 ℃ while under UV light. All scale bars represent 1 cm.

**Figure S3.** Tensile storage modulus of NOA 63 as a function of temperature. Each sample was measured after UV curing at 20, 40, 60, 80, 100, and 120 ℃.

**Figure S4.** Results of DSC measurements. The ambient temperature during UV irradiation is; (a) 20 ℃, (b) 40 ℃, (c) 60 ℃, (d) 80 ℃, (e) 100 ℃, (f) 120 ℃.

**Figure S5.** Surface roughness of substrates measured by confocal laser scanning microscope.

**Figure S6.** Time-lapse images of the shape recovery process of the modified micro-lens pattern along various substrate. ((a) Glass, (b) Paper, (c) Metal. (d) Prism. (e) Plastic (f) Back surface of Si-wafer. (g) Wood. (h) Nano-line).

**Figure S7.** Photograph of the roll-to-roll equipment used in figure 6.

**Figure S8.** Universal Testing Machine (UTM).

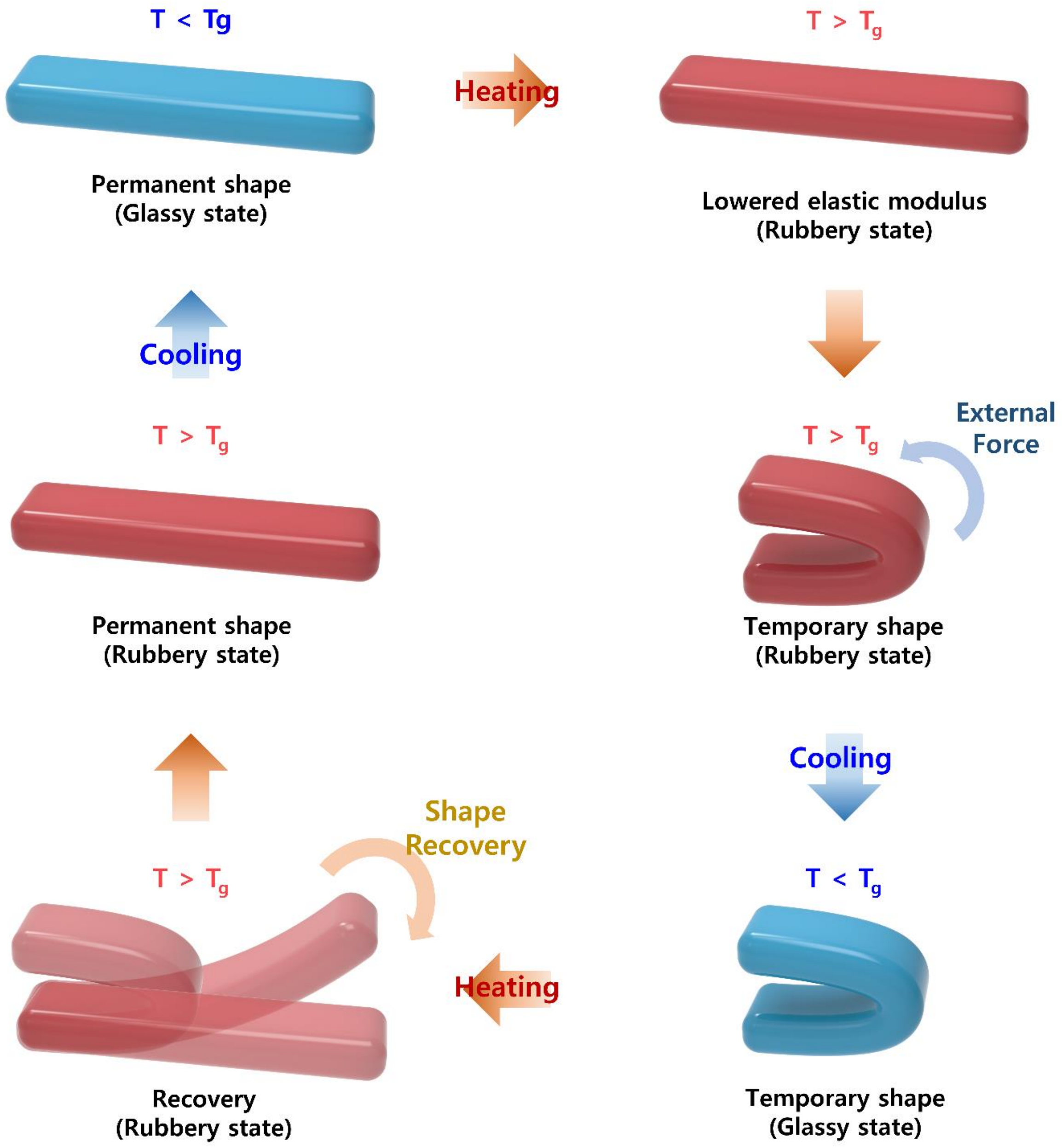


**Figure S1.** overall shape modification and recovery mechanism of shape momory polymer.

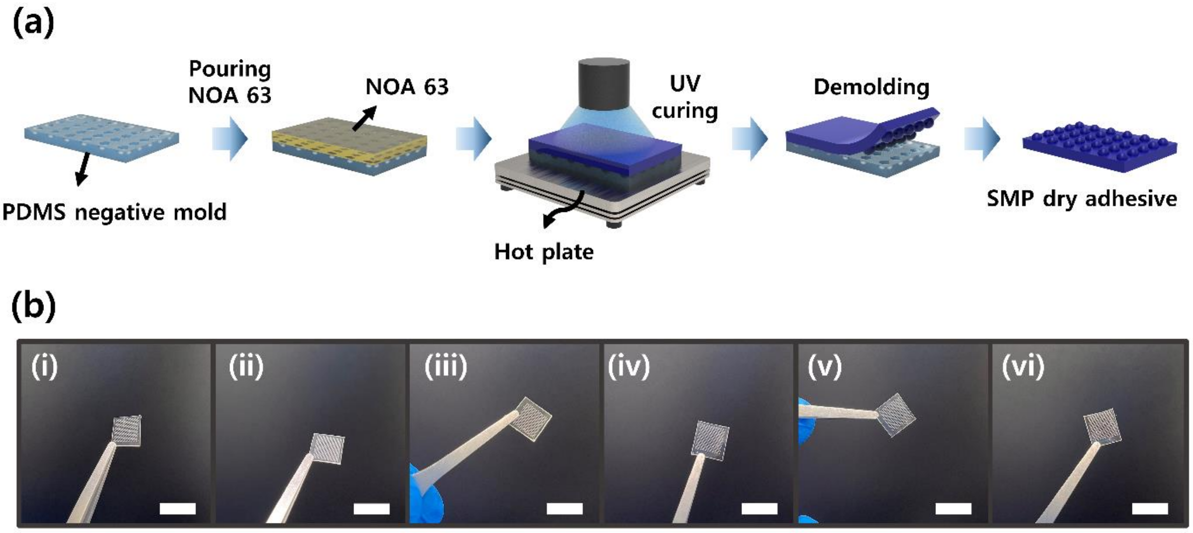


**Figure S2.** (a) Schematic illustration of the overall fabrication process of SMP dry adhesive. (b) Photo images of fabricated SMP dry adhesive. Each sample was cured at the following temperatures; (i) 20 °C, (ii) 40 °C, (iii) 60 °C, (iv) 80 °C, (v) 100 °C, (vi) 120 °C while under UV light. All scale bars represent 1 cm.

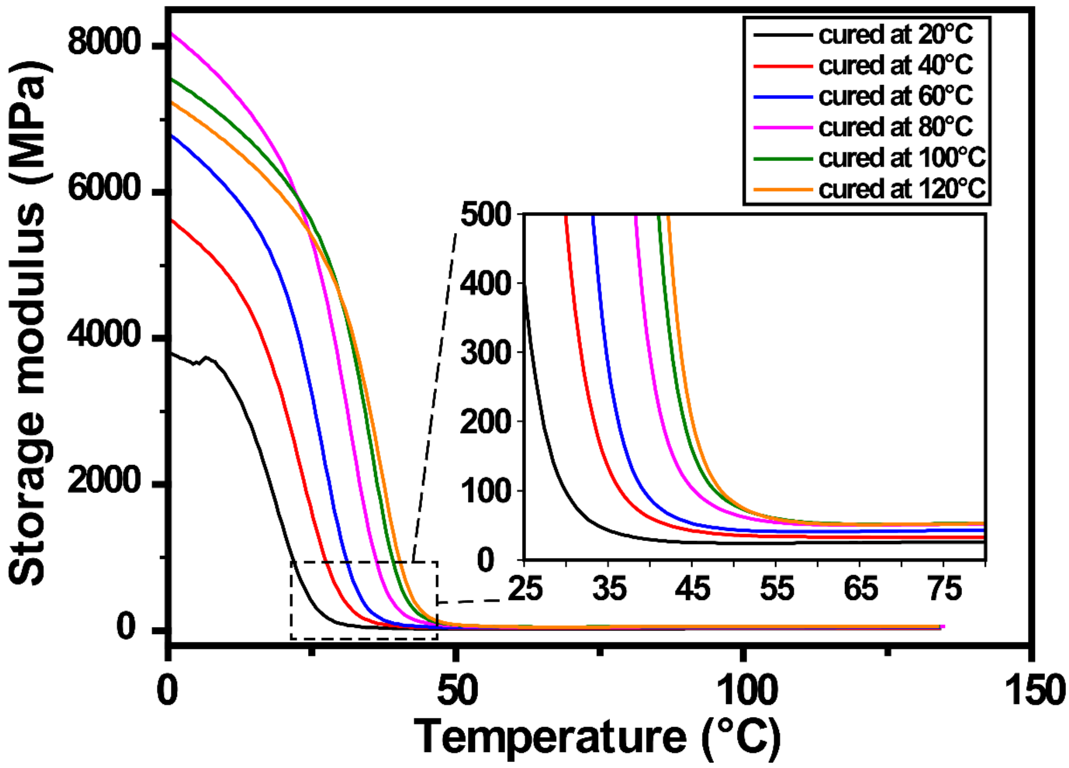


**Figure S3.** Tensile storage modulus of NOA 63 as a function of temperature. Each sample was measured after UV curing at 20, 40, 60, 80, 100, and 120 °C.

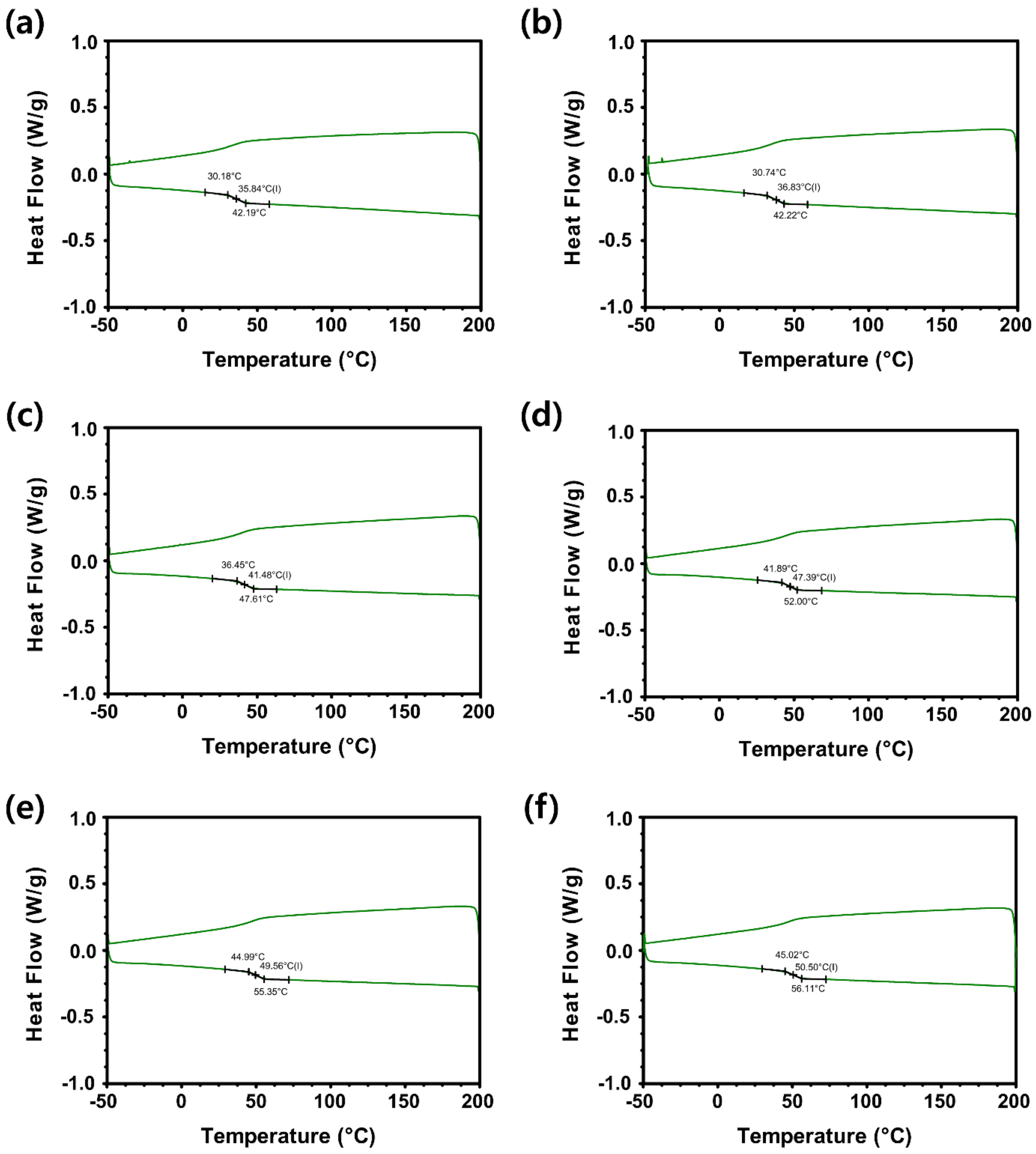


**Figure S4.** Results of DSC measurements. The ambient temperature during UV irradiation is; (a) 20 °C, (b) 40 °C, (c) 60 °C, (d) 80 °C, (e) 100 °C, (f) 120 °C.

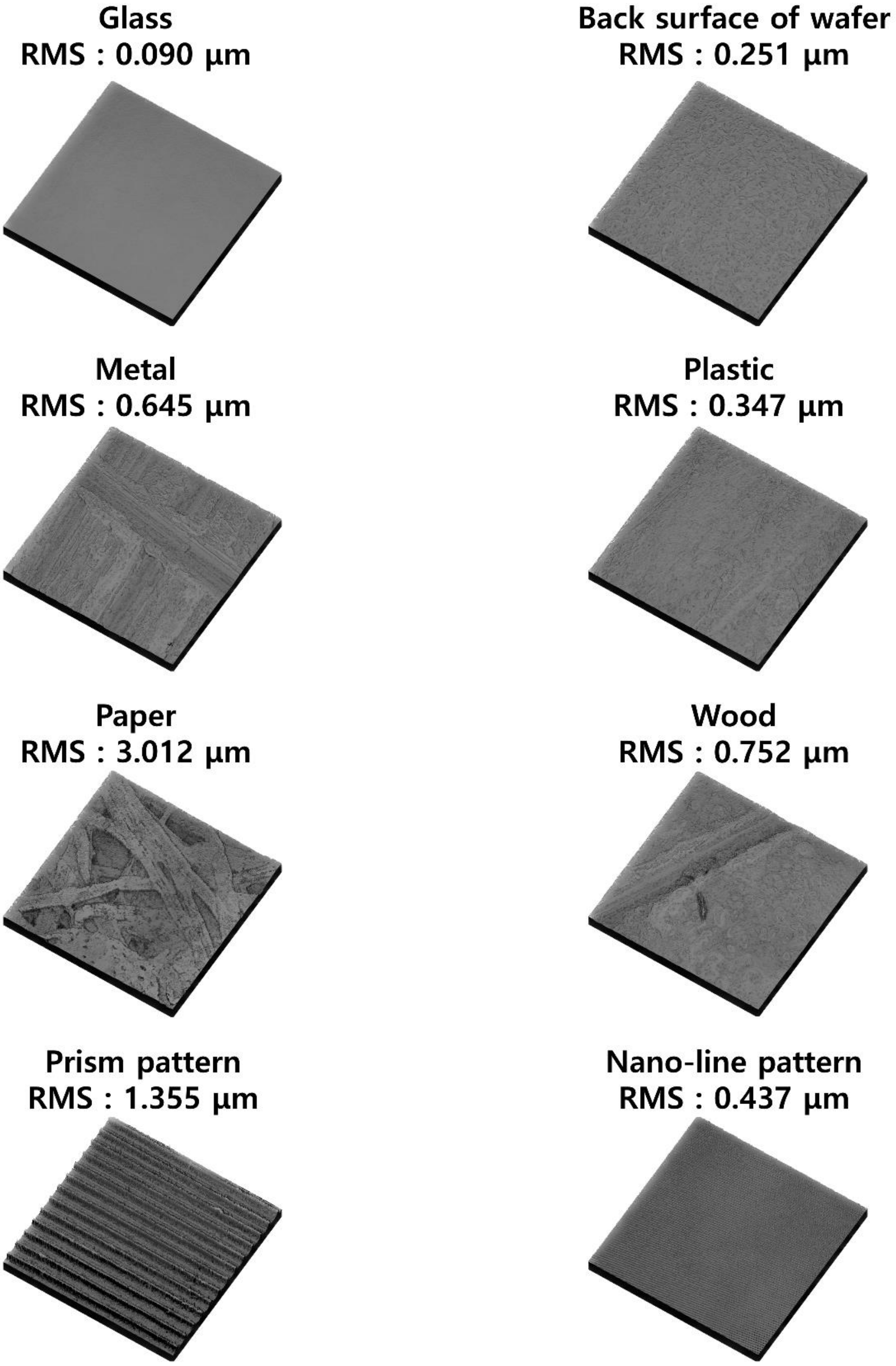


**Figure S5.** Surface roughness of substrates measured by confocal laser scanning microscope.

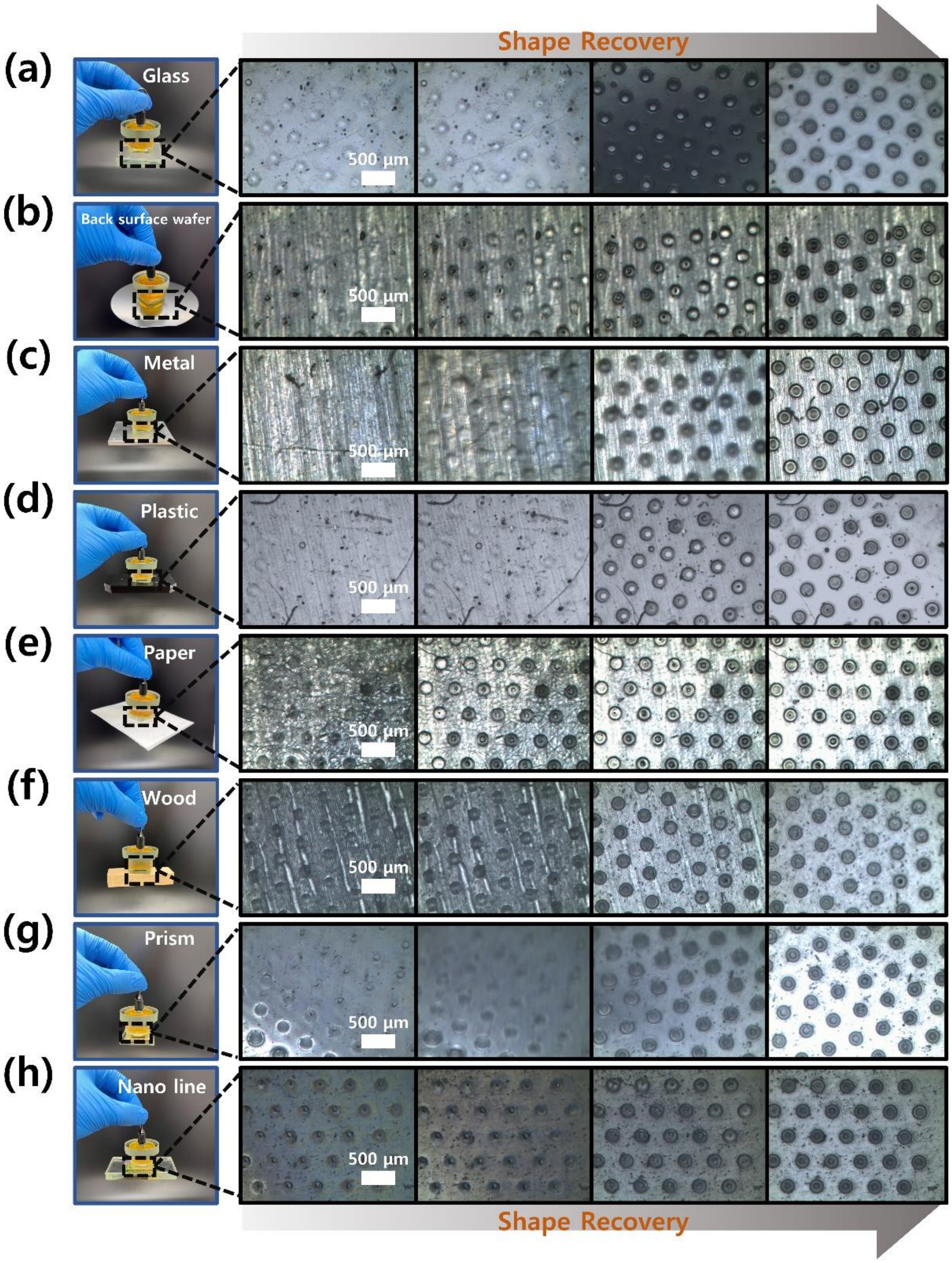


**Figure S6.** Time-lapse images of the shape recovery process of the modified micro-lens pattern along various substrate. ((a) Glass, (b) Paper, (c) Metal. (d) Prism. (e) Plastic (f) Back surface of Si-wafer. (g) Wood. (h) Nano-line).

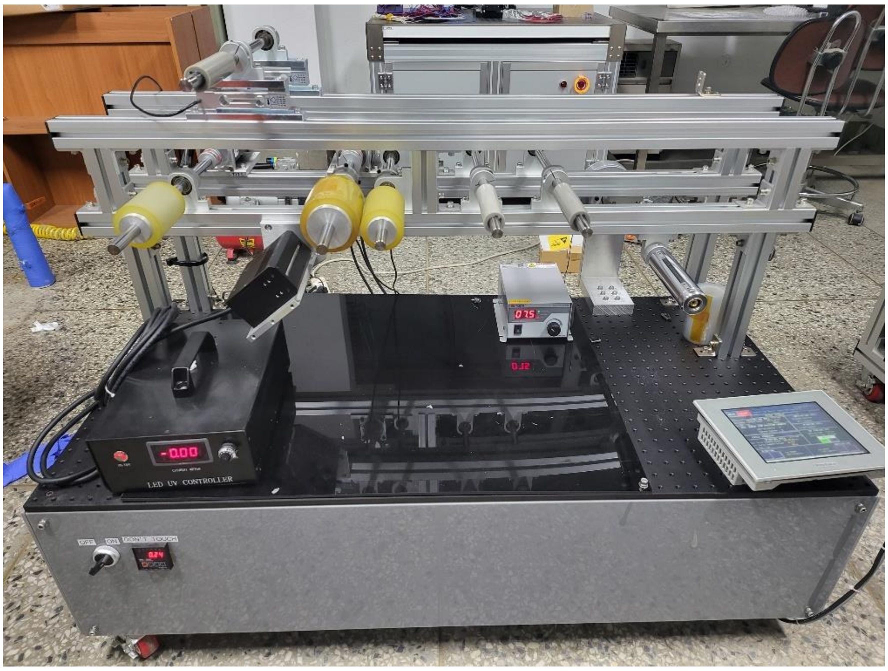


**Figure S7.** Photograph of the roll-to-roll equipment used in figure 6.

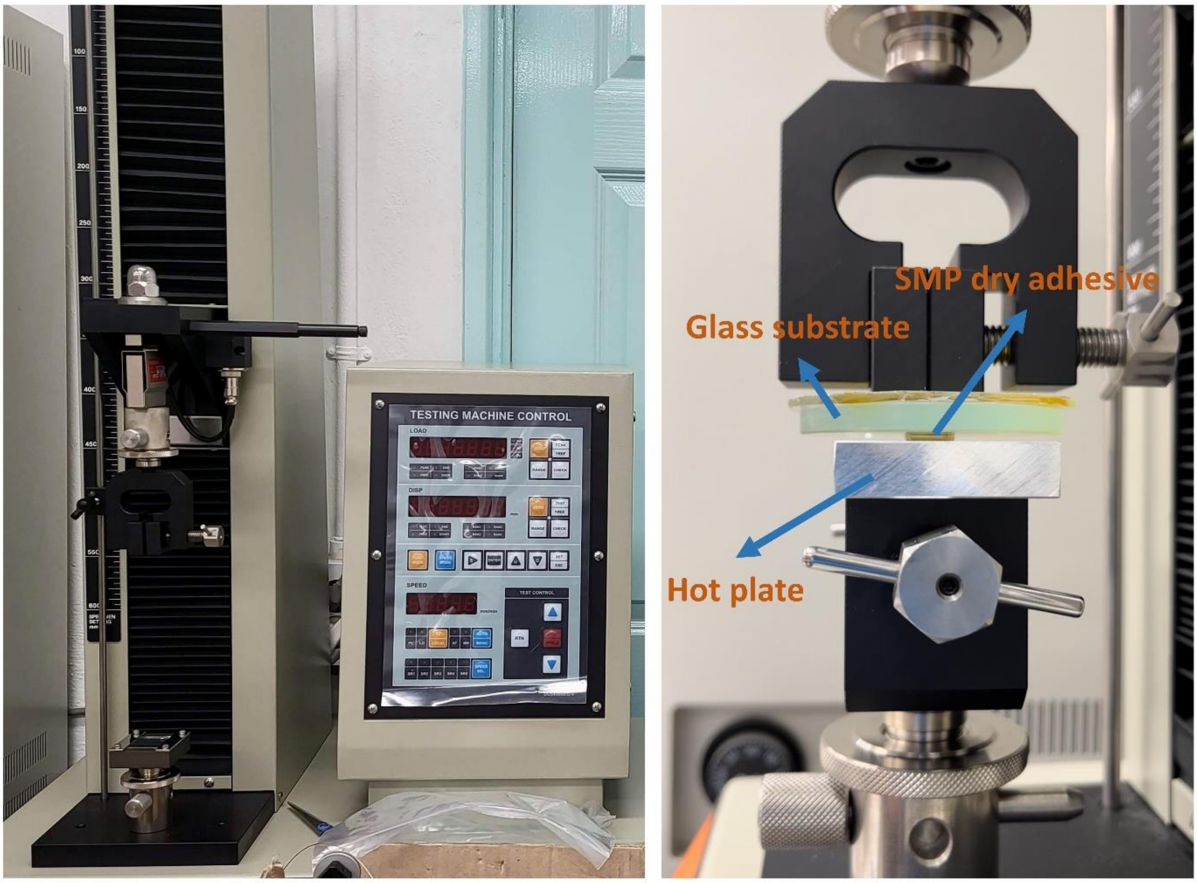


**Figure S8.** Universal Testing Machine (UTM).
